# Silent venous thromboembolism before treatment in endometrial cancer and the risk factors

**DOI:** 10.1038/sj.bjc.6604658

**Published:** 2008-09-09

**Authors:** T Satoh, K Matsumoto, K Uno, M Sakurai, S Okada, M Onuki, T Minaguchi, Y O Tanaka, S Homma, A Oki, H Yoshikawa

**Affiliations:** 1 Department of Obstetrics and Gynecology, Institute of Clinical Medicine, Graduate School of Comprehensive Human Sciences, University of Tsukuba, Ibaraki, Japan; 2 Cardiovascular Division, Institute of Clinical Medicine, Graduate School of Comprehensive Human Sciences, University of Tsukuba, Ibaraki, Japan; 3 Department of Radiology, Institute of Clinical Medicine, Graduate School of Comprehensive Human Sciences, University of Tsukuba, Ibaraki, Japan

**Keywords:** endometrial adenocarcinoma, deep vein thrombosis, pulmonary thromboembolism, plasma D-dimer

## Abstract

Venous thromboembolism (VTE) often occurs after surgery and can even occur before surgery in patients with gynaecological malignancies. We investigated the incidence of VTE before treatment of endometrial cancer and associated risk factors. Plasma D-dimer (DD) levels before initial treatment were examined in 171 consecutive patients with endometrial cancer. Venous ultrasound imaging (VUI) of the lower extremities was performed in patients with DD ⩾1.5 *μ*g ml^−1^, as the negative predictive value of DD for VTE is extremely high. For patients with deep vein thrombosis (DVT), pulmonary scintigraphy was performed to ascertain the presence of pulmonary thromboembolism (PTE). Risk factors for VTE were analysed using univariate and multivariate analyses for 171 patients. Of these, 37 patients (21.6%) showed DD ⩾1.5 *μ*g ml^−1^, 17 (9.9%) displayed DVT by VUI and 8 (4.7%) showed PTE on pulmonary scintigraphy. All patients with VTE were asymptomatic. Univariate analysis for various risk factors revealed older age, non-endometrioid histology and several variables of advanced disease as significantly associated with VTE before treatment. Obesity, smoking and diabetes mellitus were not risk factors. Multivariate analysis confirmed extrauterine spread and non-endometrioid histology as independently and significantly associated with risk of VTE. These data suggest that silent or subclinical VTE occurs before treatment in at least around 10% of patients with endometrial cancer. Risk factors for VTE before treatment might not be identical to those after starting treatment.

Deep vein thrombosis (DVT) and subsequent pulmonary thromboembolism (PTE) represent potentially lethal perioperative complications associated with major pelvic or abdominal operations (Turpie *et al*, 2002). Deep vein thrombosis is known to occur in the postoperative period or during the course of postoperative therapy in 9.8–57.1% of patients with endometrial carcinoma (Clarke-Pearson *et al*, 1983; Crandon and Koutts, 1983; Gore *et al*, 1984; von Tempelhoff *et al*, 2000). Several guidelines (Greets *et al*, 2001; Nicolaides *et al*, 2001, Committee on Practice Bulletins – Gynecology, American College of Obstetricians and Gynecologists, 2002) have focused on intra- and postoperative management for preventing venous thromboembolism (VTE). For the prevention of postoperative DVT, we usually use elastic stockings during surgery and intermittent pneumatic compression during and after surgery. However, if DVT exists before the treatment of endometrial carcinoma, such preventative measures may be ineffective or possibly dangerous for lethal PTE.

D-dimer (DD) is a degradation product of fibrin and reflects fibrin concentration. Although increased plasma DD level is generally thought to be associated with the presence of DVT, the positive predictive value of DD is 36–44%. Conversely, the negative predictive value of DD is 89–100% (Bounameaux *et al*, 1991; Harrison *et al*, 1993; Wells *et al*, 2003; Righini *et al*, 2006). We have recently reported that increased DD levels are associated with the presence of silent VTE before treatment in ovarian cancer (DD <1.5 *μ*g ml^−1^, 0 of 26 (0%); DD ⩾1.5 *μ*g ml^−1^, 18 of 46 (39%)) (Satoh *et al*, 2007). A suitable cutoff value for detecting VTE in patients with ovarian cancer seems to be 1.5 *μ*g ml^−1^, which offers 100% sensitivity and 100% negative predictive value, despite low specificity (47.2%) and low positive predictive value (38.3%). This study therefore aimed to clarify the incidence of VTE before treatment in endometrial caner patients with DD ⩾1.5 *μ*g ml^−1^ and associated risk factors.

## Materials and methods

### Patients

Between January 2004 and December 2007, a total of 171 consecutive patients with histologically confirmed endometrial carcinoma were enrolled in this study. Two patients who underwent emergent hysterectomy for vaginal bleeding were not included. Five patients for whom histological diagnosis was changed from endometrioid carcinoma to carcinosarcoma after surgery were likewise excluded from the study. All study protocols were approved by the institutional review board at University of Tsukuba Hospital, and all patients provided written informed consent to participate. Initial treatment was performed in the Department of Obstetrics and Gynecology at the University of Tsukuba Hospital. Plasma DD level was measured on the first visit to our outpatient clinic, 2–5 weeks before the initial treatment of endometrial cancer. Histology of endometrial biopsies in subjects was as follows: endometrioid carcinoma, *n*=150; adenosquamous carcinoma, *n*=4; serous carcinoma, *n*=9; clear cell carcinoma, *n*=6; mucinous carcinoma, *n*=1; and undifferentiated carcinoma, *n*=1. After surgery, histological diagnosis was changed to mixed carcinoma in three patients (endometrioid plus serous carcinoma, *n*=1; serous plus clear carcinoma, *n*=2), but diagnosis of the dominant part in these patients remained unchanged. International Federation of Gynecology and Obstetrics (FIGO) stage was as follows: stage I, *n*=101; stage II, *n*=18; stage III, *n*=32; and stage IV, *n*=20. However, we used presumed stage as evaluated by imaging studies such as computed tomography (CT) and magnetic resonance imaging (MRI) to analyse risk factors of VTE in this study. Presumed stage was as follows: stage I, *n*=107; stage II, *n*=13; stage III, *n*=29; and stage IV, *n*=22. Surgical or pathological FIGO staging resulted in upstaging in 21 cases and downstaging in 17 cases. Mean patient age was 58.1 years (range, 19–84) and mean body mass index (BMI) was 25.0 kg/m^2^ (range, 16.8–43.4).

### Measurement of plasma DD level

Peripheral blood samples were collected from all patients before treatment and DD levels were measured. Blood samples were drawn from an antecubital vein by atraumatic puncture into plastic tubes using a two-tube technique, discarding the first 4 to 5 ml. Whole blood was anticoagulated by the addition of nine volumes to one volume of 0.11 mol l^−1^ sodium citrate solution, then centrifuged at 3000 r.p.m. for 10 min. Citrated plasma was then removed and frozen at −20°C up to 3 days before assessment.

Plasma DD level was measured using STA-Liatest D-Di latex (Diagnostica Stago, Asniēres, France) sensitised with anti-DD mouse monoclonal antibody to induce a latex coagulation reaction, then turbidity was quantified using a spectrophotometer. The cutoff value for plasma DD in this method is 0.5 *μ*g ml^−1^.

### Detection of DVT

All patients underwent comprehensive imaging studies using CT and MRI to detect abdominal tumour extension and thrombus in iliac veins and the inferior vena cava. Venous ultrasound imaging (VUI) was performed to detect DVT only in patients with elevated DD level of ⩾1.5 *μ*g ml^−1^. Ultrasonography was performed using an ATL HDI5000 system (Philips Medical Systems, Bothell, WA, USA) equipped with a 3–7.5 MHz transducer. Power, pulse repetition frequency and wall thump filter settings were adjusted for venous vascular studies. Iliac, femoral, great saphenous, popliteal, peroneal, post-tibial and soleal veins were evaluated bilaterally. Iliac and femoral veins were assessed in a supine position and other veins were assessed in an upright position. All veins were imaged on transverse and long axis views. Venous lumina were observed while searching for thrombus by manual compression with transducer and colour Doppler imaging. For the evaluation of intrapelvic veins, reactions during the Valsalva manoeuvre were also observed. No reaction during the Valsalva manoeuvre was considered to represent suspected proximal venous flow disturbance, and intrapelvic DVT was diagnosed based on the results of enhanced CT. However, no patients displayed intrapelvic DVT in this study.

### Pulmonary perfusion scintigraphy

All patients with DVT detected by VUI were examined by pulmonary perfusion scintigraphy using Tc-99 to evaluate the presence of PTE.

### Management of patients with VTE before treatment

Anticoagulant therapy was started using unfractionated heparin immediately after diagnosis of VTE. Therapy was continued until initial treatment, comprising surgery or chemotherapy, for all patients with VTE. We always administered anticoagulant therapy after surgery in these patients. In addition, one of the following managements was selected in some patients: placement of an inferior vena cava filter (IVCF) before upfront surgery; or neoadjuvant chemotherapy (NAC) followed by surgery.

Inferior vena cava filter placement was used before upfront surgery to prevent lethal PTE in patients with DVT in proximal veins, such as iliac and femoral veins or floating DVT in peripheral veins. Neoadjuvant chemotherapy followed by surgery was selected for patients with apparent stage III/IV endometrial cancer and chemosensitive tumour histology, such as serous or endometrioid type as estimated on biopsy and excessively increased CA125 level. We administered a paclitaxel and carboplatin (TC) regimen comprising paclitaxel (175 mg m^−2^ infused over >3 h) and AUC 6 of carboplatin.

We also used pelvic irradiation, chemotherapy using a TC regimen or sequential chemoradiation therapy (TC regimen followed by pelvic and/or paraaortic radiation) as postoperative therapy for high-risk patients with stage Ic-IV endometrial cancer.

For patients with lower DD levels (<1.5 *μ*g ml^−1^) and patients with higher DD levels (⩾1.5 *μ*g ml^−1^) but without VTE, compression stockings and intermittent pneumatic compression were used during and after surgery to prevent postoperative VTE. Low-molecular-weight heparin was also administered before and after surgery in patients with BMI ⩾28 kg/m^2^ and patients who had been administered anticoagulative medication due to a past history of cerebral infarction or atrial fibrillation.

## Results

### Plasma level of DD and incidence of VTE before treatment

Plasma DD level was above the reference value (0. 5 *μ*g ml^−1^) in 74 of 171 patients with endometrial cancer (43.3%). Thirty-seven (21.6%) patients showed DD ⩾1.5 *μ*g ml^−1^. Subsequent VUI and PS revealed DVT in 17 patients (9.9%) and PTE in 8 patients (4.7%) (DVT alone, *n*=9; DVT plus PTE, *n*=8) before initial treatment. The most proximal location of DVT in these patients was the peroneal vein in eight patients, popliteal vein in two patients and soleal vein in seven patients. Mean DD level in 17 patients with VTE was 7.75 *μ*g ml^−1^ (range, 1.7–21.6). D-dimer levels were associated with the incidence of VTE (1.5–2.9 *μ*g ml^−1^, 3 out of 16 (13.6%); 3.0–4.5 *μ*g ml^−1^, 3 out of 6 (50.0%); and >4.5 *μ*g ml^−1^, 11 out of 15 (73.3%); *P*-value for trend=0.009). All patients with VTE were asymptomatic when VTE was identified.

As VUI was performed only in patients with DD ⩾1.5 *μ*g ml^−1^, sensitivity, specificity and negative predictive values were not able to be evaluated. The positive predictive value of DD using 1.5 *μ*g ml^−1^ as the cutoff level for silent VTE was 46.0% (17/37).

### Risk factors for VTE before treatment

Table 1 shows relative risks for VTE according to personal characteristics, direct invasion, tumour extension, histology and tumour markers. Study variables determined before initial treatment were used in this analysis, with tumour extension (presumed stage) and myometrial invasion from imaging studies, histology from endometrial biopsy and tumour markers before treatment. Among these variables, older age, large tumour size in the uterine cavity, deep myometrial invasion, extrauterine spread, massive ascites, ovarian metastasis, paraaortic lymph node swelling, peritoneal dissemination, non-endometrioid histology, clear cell histology and increased tumour markers (CA125, CA19-9 and CEA) were significantly associated with VTE before treatment in univariate analysis. Subsequently, we performed the multivariate analysis using several significant variables in the univariate analysis. In the multivariate analysis, we focused on the following four representative variables, because there were apparent correlations among some variables (data not shown). ‘Tumour extention (extrauterine spread)’ was selected out of various significant variables representing tumour extension or metastasis including tumour markers. Similarly, we selected ‘myometrial invasion’ over ‘tumour size in the uterine cavity’ as a factor representing direct invasion and ‘non-endometrioid histology’ over ‘clear cell histology’ as a factor representing histology. Multivariate analysis for age, extrauterine spread, myometrial invasion and non-endometrioid histology confirmed extrauterine spread and non-endometrioid histology as independently and significantly associated with the risk of VTE before treatment (Table 2).

### Management of VTE before treatment

Anticoagulant therapy using unfractionated heparin was administered before initial treatment for all 17 patients with VTE and after surgery for 14 patients who underwent primary or interval surgery. Three patients underwent no surgery because two patients displayed disease progression during NAC and the remaining one patient had severe heart disease. In addition to anticoagulant therapy, the placement of IVCF before upfront surgery was used for one patient with floating DVT, and NAC was performed for five patients. In summary, we managed patients with VTE before treatment as follows: (1) anticoagulant therapy alone in 11 patients; (2) upfront surgery with placement of IVCF in 1 patient; and (3) NAC followed by surgery in 5 patients.

### VTE after commencement of treatment

None of the 17 patients with silent VTE before treatment developed clinical manifestations of VTE after the commencement of treatment, whereas 7 of the remaining 154 patients (4.6%) developed VTE (DVT alone, *n*=4; DVT plus PTE, *n*=3), after surgery in 3 patients and during chemotherapy in 4 patients. Of these seven patients, four patients had asymptomatic VTE (DVT alone, *n*=3; DVT plus PTE, *n*=1), and VTE was identified on routine CT and subsequent PS after surgery or during chemotherapy. Three of the seven patients showed DD ⩾1.5 *μ*g ml^−1^ before treatment, although DVT was not detected by VUI.

## Discussion

This study detected silent DVT and DVT plus PTE before treatment in 9.9 and 4.7%, respectively, of patients with endometrial cancer. These incidences appear lower than 25.0 and 11.1%, respectively, in ovarian cancer reported in our previous paper (Satoh *et al*, 2007), although incidences in endometrial cancer were higher than the 5.2 and 1.2% in cervical cancer, respectively (unpublished data). As for DD level, 21.6% showed levels ⩾1.5 *μ*g ml^−1^ in endometrial cancer, suggesting a lower percentage than the 63.9% in ovarian cancer and higher than the 14.5% in cervical cancer (unpublished data). However, several studies have documented higher incidence of postoperative DVT and PTE in endometrial cancer than in ovarian cancer (Clarke-Pearson *et al*, 1983; Crandon and Koutts, 1983; Gore *et al*, 1984; Suzuki *et al*, 2005). Incidence of silent VTE before surgery is unlikely to account for the high incidence of VTE after surgery in endometrial cancer.

Univariate and multivariate analyses revealed extrauterine spread and non-endometrioid histology as independently and significantly associated with the risk of VTE before treatment in endometrial cancer. Many variables regarding advanced disease were identified as significant risk factors for VTE before treatment in endometrial cancer, whereas our recent study revealed that various risk factors regarding advanced disease other than massive ascites were not significant risk factors for VTE before treatment in ovarian cancer. As for VTE after surgery or during treatment, a considerable number of previous reports have indicated advanced disease as a significant risk factor for VTE in gynaecological malignancies (Dubuc-Lissoir *et al*, 1999; Agnelli *et al*, 2006; Wang *et al*, 2006).

Among non-endometrioid histologies, clear cell histology seems to represent the highest risk factor (clear cell histology, 4 out of 6 (66.7%); non-clear cell histology, 4 out of 15 (26.7%)). We have recently reported clear cell histology as a strong risk factor for VTE before treatment in ovarian cancer (Satoh *et al*, 2007), and clear cell carcinoma shows significantly stronger expression of tissue factor, a major factor in the procoagulant activities of cancer cells, as compared with non-clear cell carcinoma (Uno *et al*, 2007). Several published reports support the association between clear cell histology and VTE in ovarian cancer (Goff *et al*, 1996; Recio *et al*, 1996). These reports have postulated that patients with clear cell histology display a significantly higher incidence (11–42%) of VTE as compared with those with non-clear cell histology in the immediate postoperative period and during primary chemotherapy. Goff *et al* (1996) reported two ovarian cancer patients with clear cell histology displaying DVT as a presenting symptom before surgery. We have also reported an endometrial cancer patient with clear cell histology showing VTE before treatment (Toyoda *et al*, 2005). The same histology among different organs of mullerian origin, such as the cervix, endometrium, ovaries and fallopian tubes, is known to have some common genetic or biological characteristics (Vang *et al*, 2001). The predisposition to VTE might be one common biological characteristic of clear cell histology in the endometrium and ovary.

Venous thromboembolism is one of the major postoperative complications of endometrial cancer. Obesity, diabetes mellitus and high oestrogen state, which often accompany endometrial cancer, all represent risk factors for VTE (Ageno *et al*, 2008). In addition, smoking, immobility, autoimmune disease, varicosity, giant pelvic tumours, congestive cardiac failure and hyperlipidaemia are known risk factors for VTE after surgery. However, silent VTE before treatment was not associated with obesity, smoking or diabetes mellitus in this study (Table 1). In the univariate and multivariate analyses of risk factors for VTE after the commencement of treatment in 154 patients without VTE before treatment, DD ⩾1.5 *μ*g ml^−1^ before treatment, obesity (BMI >35 kg/m^2^) and FIGO stage III/IV were all independent and significant risk factors (data not shown). Advanced disease, extrauterine spread or FIGO stage III/IV and DD ⩾1.5 *μ*g ml^−1^ before treatment were risk factors for VTE both before and after starting treatment, whereas non-endometrioid histology was a risk factor for VTE before treatment and obesity was a risk factor for VTE after starting treatment.

In the guidelines for preventing VTE following general surgery reported at the sixth American College of Chest Physicians Consensus Conference (Greets *et al*, 2001), patients with gynaecologic malignancies are classified as the highest risk group. These guidelines focused on the prevention of postoperative VTE and recommended the use of elastic stockings and intermittent pneumatic compression during and after surgery, and anticoagulant therapy after surgery in patients with gynaecologic malignancies. However, this study and our recent study revealed that patients may have silent or subclinical VTE even before treatment in the presence of endometrial or ovarian cancers. Given that VTE before treatment may represent the highest risk for VTE after the commencement of treatment unless special management is performed, preoperative assessment of VTE may be important for reducing the incidence of VTE after starting treatment.

In conclusion, the measurement of plasma DD level and subsequent VUI revealed that silent or subclinical VTE occurs before surgery in at least around 10% of patients with endometrial cancer, although it might be presumed that if all patients had had VUI, slightly more VTE would be found than reported here. Detection of VTE before the treatment and management of VTE may contribute to preventing postoperative VTE. However, VTE can often occur after the commencement of treatment in endometrial cancer patients without VTE before treatment, particularly among those with risk factors such as DD ⩾1.5 *μ*g ml^−1^ before surgery, obesity, advanced stage or invasive surgery. We recommend the assessment and management of VTE before and after starting treatment of endometrial cancer as follows: (1) measurement of DD level should be considered before treatment in all patients. (2) Venous ultrasound imaging should be performed before treatment in patients with DD ⩾1.5 *μ*g ml^−1^. (3) If VTE was found before cancer treatment, heparin treatment should be started immediately. (4) For the prevention of VTE after starting treatment, heparin should be used after surgery or during chemotherapy at least for patients with DD ⩾1.5 *μ*g ml^−1^ before treatment, obesity or advanced stage in addition to patients with VTE before treatment. Further clarification of the risk factors for VTE before and after commencement of treatment is needed to prevent VTE in endometrial cancer.

## Figures and Tables

**Table 1:** Relative risks (ORs) of venous thromboembolism according to personal characteristics, tumour extension, direct invasion, histology and tumour markers

**Characteristics**	**Incidence rate**	**OR (95% CI)**	***P*-value**
*Age (years)*			
<60	6/103 (5.8%)	Reference	
⩾60	11/68 (16.2%)	3.12 (1.09–8.89)	0.03
			
*Body mass index (kg/m*^*2*^)			
<25	12/103 (11.7%)	Reference	
⩾25	5/68 (7.4%)	0.61 (0.20–1.79)	0.88
			
*Smoking*			
Non-smoker	15/158 (36.8%)	Reference	
Smoker	2/13 (100%)	1.77 (0.35–8.57)	0.49
			
*Menopause*			
No	4/38 (10.5%)	Reference	
Yes	13/133 (9.7%)	0.92 (0.28–3.01)	0.52
			
*Parity*			
<3	11/131 (8.4%)	Reference	
⩾3	6/40 (15.0%)	1.93 (0.66–5.59)	0.18
			
*Diabetes mellitus*			
No	13/144 (9.0%)	Reference	
Yes	4/27 (100%)	1.75 (0.53–5.85)	0.27
			
*Anticoagulative medication for brain or heart disease*			
No	17/166 (10.2%)	Reference	
Yes	0/5 (0.0%)	0	1.00
			
*Tumour size in uterine cavity (MRI) (mm)*			
<60	6/134 (4.5%)	Reference	
⩾60	11/26 (29.7%)	9.03 (3.06–26.6)	<0.0001
			
*Myometrial invasion (MRI)*			
⩽1/2	7/120 (5.8%)	Reference	
>1/2	10/51 (19.6%)	3.94 (1.41–11.0)	0.009
			
*Tumour extension (CT/MRI)*			
Localised to the uterus	3/120 (2.5%)	Reference	
Extrauterine spread	14/51 (27.5%)	14.8 (4.02–54.2)	<0.0001
			
*Plevic LN swelling >10 mm (CT)*			
Absent	11/141 (7.8%)	Reference	
Present	6/30 (20.0%)	2.95 (0.997–8.75)	0.053
			
*Paraaortic LN swelling >10 mm (CT)*			
Absent	10/150 (6.7%)	Reference	
Present	7/21 (33.3%)	7.00 (2.30–21.3)	0.001
			
*Massive ascites (CT)* a			
Absent	14/165 (8.48%)	Reference	
Present	3/6 (50%)	10.8 (1.99–58.5)	0.01
			
*Ovarian metastasis (MRI)*			
Absent	12/158 (7.6%)	Reference	
Present	5/13 (38.5%)	7.60 (2.15–26.9)	0.004
			
*Peritoneal dissemination (CT)*			
Absent	10/154 (6.5%)	Reference	
Present	7/17 (53.9%)	10.1 (3.16–32.1)	0.0003
			
*Distant metastasis except for peritoneal dissemination (CT or physical examination)*			
Absent	15/166 (9.0%)	Reference	
Present	2/5 (40.0%)	6.7 (1.04–43.4)	0.08
			
*Histology*			
Endometrioid carcinoma	9/150 (6.0%)	Reference	
Non-endometrioid carcinoma	8/21 (38.1%)	9.64 (4.46–46.0)	0.0002
Serous carcinoma	3/9 (33.3%)	7.83 (1.68–36.6)	0.02
Clear cell carcinoma	4/6 (66.7%)	31.3 (5.05–195)	0.0004
			
*CA125 (ULN: 35 U ml*^*−1*^)			
⩽35	4/114 (3.5%)	Reference	
>35	13/57 (22.8%)	8.13 (2.51–26.3)	0.0002
⩽70	6/133 (4.5%)	Reference	
>70	11/38 (29.0%)	8.62 (2.93–25.3)	<0.0001
			
*CA19-9 (ULN:37 U ml*^*−1*^)			
⩽37	8/95 (8.4%)	Reference	
>37	8/62 (50.0%)	1.61 (0.57–4.54)	0.26
⩽74	8/118 (6.8%)	Reference	
>74	8/39 (20.5%)	3.55 (1.23–10.2)	0.02
			
*CEA (ULN:5 ng ml*^*−1*^)			
⩽5	11/132 (8.3%)	Reference	
>5	6/23 (26.1%)	3.88 (1.27–11.9)	0.02
<10	14/147 (9.5%)	Reference	
⩾10	3/8 (37.5%)	5.70 (1.23–26.4)	0.04

95% CI=95% confidence interval; LN=lymphonode; OR=odds ratio; ULN=upper limits of normal.

a Massive ascites was defined as centralisation detected by CT in this study.

**Table 2:** The multivariate analysis for the four representative risk factors of venous thromboembolism

**Characteristics**	**VTE**	**RR (95% CI)**	***P*-value**
*Age (years)*			
<60	6/103 (5.8%)	Reference	
⩾60	11/68 (16.2%)	1.82 (0.47–7.34)	0.38
			
*Tumour extension (CT/MRI)*			
Localised to the uterus	3/120 (2.5%)	Reference	
Extrauterine spread	14/51 (27.5%)	14.4 (3.24–83.2)	0.001
			
*Histology*			
Endometrioid carcinoma	9/150 (6.0%)	Reference	
Non-endometrioid carcinoma	8/21 (38.1%)	4.61 (1.20–18.0)	0.03
			
*Myometrial invasion (MRI)*			
⩽1/2	7/120 (5.8%)	Reference	
>1/2	10/51 (19.6%)	0.50 (0.13–2.52)	0.50

95% CI=95% confidence interval; RR=risk ratio; VTE=venous thromboembolism.
